# Editorial: Highlights in women's mental health 2021/22

**DOI:** 10.3389/fgwh.2023.1323318

**Published:** 2023-11-08

**Authors:** Lauren M. Osborne, Reem Kais Jan, Jayashri Kulkarni

**Affiliations:** ^1^ Department of Obstetrics and Gynecology, Weill Cornell Medicine, Cornell University, New York, NY, United States; ^2^ Department of Pharmacology, Mohammed Bin Rashid University of Medicine and Health Sciences, Dubai, United Arab Emirates; ^3^ Department of Psychiatry, Monash University, Melbourne, VIC, Australia

**Keywords:** women’s mental health, insomnia, postpartum, healthcare utilization, breastfeeding

**Editorial on the Research Topic** Highlights in women’s mental health 2021/22

The Research Topic *Highlights in Women's Mental Health 2021/22* was designed to showcase a selection of high-impact articles across the field of women's mental health—not grouped by content, but rather representative of the breadth of research in this growing field. Those of us who do research in this field may use different terms to refer to our expertise, depending on the particular niche we occupy (for example, perinatal psychiatry is one such niche). The broad term “women's mental health” represents a multidisciplinary field focused on the influence of psychopathology and treatment on three different domains: (1) female reproductive cycles (2) female sex and (3) female gender. Work on female reproductive cycles includes biological and psychosocial research at times of reproductive hormonal transition (for example, menarche, the premenstrual period, pregnancy, postpartum, and perimenopause). Work on female sex includes topics such as brain sexual dimorphism, female-specific comorbidities, and pharmacokinetic sex differences. Work on female gender includes topics such as gender roles and gender-linked trauma. Each of these areas is undergoing transition as definitions of gender change in our culture, with reproductive hormonal transitions now including people who do not identify as women and may have different and broader hormonal transitions, and gender including people who do not identify on a male/female binary.

This Research Topic consists of a collection of papers that represent this broad field. Two of our published papers center on psychopathology related to reproductive cycles. The first, by Verma et al., is a protocol paper for a randomized controlled trial of an intervention for insomnia that compared cognitive behavioral therapy to light/dark therapy and to a control condition for individuals in the later postpartum (between 4 and 12 months). The two treatment conditions were centered in evidence-based solutions that we know can be helpful outside the perinatal period, and individuals assigned to each condition were supported by telephone calls from perinatal psychotherapists. Sleep research in postpartum women is surprisingly scarce, given the prevalence of sleep disturbance in this population, and rarely focused on the later postpartum; this rigorously designed trial therefore promises to shed new light on a common and impairing problem. The second paper in this area, by Kimmel et al., combined data from three continents to offer new insights into postpartum affective disorders. The authors pointed out that, while treatment algorithms for new-onset postpartum psychosis, a rare phenomenon, have been established, there has been less attention paid to clinical features and best treatment practices for those suffering in the postpartum from major depressive disorder with psychosis, or from manic or mixed episodes without psychosis. To tackle this important issue, they combined data from three inpatient units specifically designed to treat severe postpartum disorders, in India, the Netherlands, and the United States. They compared diagnosis and treatment across the three centers and found vast differences in prevalence rates of the three conditions as well as substantial differences in treatment choices.

Our Research Topic also included two papers addressing healthcare utilization and health practices among women with severe mental disorders. Kavanagh et al. addressed the important issue of personality disorders, comparing healthcare utilization patterns among women with a mental health disorder not including a personality disorder, those with a mental health disorder including a personality disorder, and healthy controls. They enrolled over 600 women and compared utilization of both general medical and mental health services. They found that women with mental health disorders including personality disorder had more encounters with non-mental health services than did both of the other groups, and that this was particularly pronounced at younger ages. This work provides important information for healthcare practitioners, who should be alert to this different usage pattern and to the particular needs of this population. In the next paper, Baker et al. addressed the important issue of differences in health-related practices among women with severe mental illness. They examined infant feeding intentions and practices in a cohort of women admitted for inpatient care during the first postpartum year. They found that the vast majority of women intended to breastfeed, and that 75% of those who intended to breastfeed did so in some capacity—but that the rate of breastfeeding initiation was substantially lower than that in the general population. They also found that a significant minority of women were given erroneous advice by a healthcare practitioner about the compatibility of breastfeeding with their psychiatric medications, and that this resulted in women not breastfeeding or stopping earlier than intended. This study highlights the lack of knowledge and support surrounding breastfeeding in women with mental illness, and argues for better provider training.

Finally, this Research Topic also includes two papers on aspects of violence, though from two very different lenses. Klimovich-Mickael et al. addressed trends in anger and physical aggression among women in Russia during the COVID-19 lockdown. They surveyed women in the general population (so without known mental disorders) to examine rates of anxiety, depression, and physical aggression during the lockdown. All participants were female homemakers who were participating in an online fitness platform. While they did not have a comparison group for anxiety and depression (which were present in at least mild form for 77.4% and 54.8% of their population, respectively), they were able to compare rates of physical and verbal aggression, anger, and hostility against those collected previously by the designer of the scale used (Buss-Perry Aggression Questionnaire), and found that all elements but hostility as well as the total score were significantly elevated in their population. While these data do not allow direct comparisons to this population in the absence of lockdown, they do indicate high rates of psychological distress in that period. St. John and Walmsley tackle another side of violence and aggression in their review of the latest treatment interventions to improve mental health following gender-based violence in low and middle-income countries. Their review highlights an impressive rate of innovation; they found 16 new interventions designed in the past five years to tackle such mental-health sequelae. Only one of these interventions was successfully deployed in a healthcare setting, and interventions deployed in conflict settings were not found to be effective. Group-based interventions led by medical professionals had low engagement, and therefore unclear efficacy. Community-based interventions, however, showed clear promise. The authors were able to identify several factors associated with success across all types of interventions: (1) clear assessment of all psychiatric disorders prior to treatment, (2) frequency of care provision, and (3) appropriateness of setting. This review can pave the way for the design of future interventions to tackle this important issue.

Altogether, this Research Topic demonstrates the breadth and diversity of research in women's mental health across the globe, setting the stage for further in-depth studies in each of these areas.

